# Construction of cancer- associated fibroblasts related risk signature based on single-cell RNA-seq and bulk RNA-seq data in bladder urothelial carcinoma

**DOI:** 10.3389/fonc.2023.1170893

**Published:** 2023-04-14

**Authors:** Yunxun Liu, Jun Jian, Ye Zhang, Lei Wang, Xiuheng Liu, Zhiyuan Chen

**Affiliations:** ^1^ Department of Urology, Renmin Hospital, Wuhan University, Wuhan, China; ^2^ Institute of Urologic Disease, Renmin Hospital, Wuhan University, Wuhan, China

**Keywords:** cancer-associated fibroblasts, bladder urothelial carcinoma, tumor microenvironment, immunotherapy, single-cell RNA-seq, prognosis

## Abstract

**Background:**

The ability of cancer-associated fibroblasts (CAFs) to encourage angiogenesis, tumor cell spread, and increase treatment resistance makes them pro-tumorigenic. We aimed to investigate the CAF signature in Bladder urothelial carcinoma (BLCA) and, for clinical application, to build a CAF-based risk signature to decipher the immune landscape and screen for suitable treatment BLCA samples.

**Methods:**

CAF-related genes were discovered by superimposing CAF marker genes discovered from single-cell RNA-seq (scRNA-seq) data taken from the GEO database with CAF module genes discovered by weighted gene co-expression network analysis (WGCNA) using bulk RNA-seq data from TCGA. After identifying prognostic genes related with CAF using univariate Cox regression, Lasso regression was used to build a risk signature. With microarray data from the GEO database, prognostic characteristics were externally verified. For high and low CAF-risk categories, immune cells and immunotherapy responses were analyzed. Finally, a nomogram model based on the risk signature and prospective chemotherapeutic drugs were examined.

**Results:**

Combining scRNA-seq and bulk-seq data analysis yielded a total of 124 CAF-related genes. LRP1, ANXA5, SERPINE2, ECM1, RBP1, GJA1, and FKBP10 were the seven BLCA prognostic genes that remained after univariate Cox regression and LASSO regression analyses. Then, based on these genes, prognostic characteristics were created and validated to predict survival in BLCA patients. Additionally, risk signature had a strong correlation with known CAF scores, stromal scores, and certain immune cells. The CAF-risk signature was identified as an independent prognostic factor for BLCA using multifactorial analysis, and its usefulness in predicting immunotherapy response was confirmed. Based on risk classification, we projected six highly sensitive anticancer medicines for the high-risk group.

**Conclusion:**

The prognosis of BLCA may be accurately predicted using CAF-based risk signature. With a thorough understanding of the BLCA CAF-signature, it might be able to explain the BLCA patients’ response to immunotherapy and identify a potential target for BLCA treatment.

## 1 Introduction

BLCA is among the most common cancers globally and is also one of the leading causes of cancer death. Incidence and mortality rates are rising in some Eastern European and developing countries (1). By 2030, it is predicted that the number of cases and deaths from bladder cancer in China will continue to increase (2). TNM staging is the major method for determining the prognosis of BLCA patients. TNM staging, however, is no longer entirely competent in the clinical context to identify individuals with varied prognoses of BLCA, suggesting that additional variables impact long-term outcomes. As a result, it is critical to create innovative multigene signatures capable of accurately predicting BLCA outcomes and immunotherapy response.

Tumor epithelial cells coexist with a variety of non-tumor mesenchymal cells that together form the tumor microenvironment (TME). Among the multiple stromal cell types in the TME, cancer-associated fibroblasts (CAFs) are a major component of many cancer types, including breast, colon, pancreatic, and prostate cancers (3). In addition to altering the extracellular matrix (ECM), CAF also interact with other TME components by secreting a variety of cytokines and growth factors. This results in an immunosuppressive TME and the development of immune evasion in cancer cells (4). It is accomplished primarily by increasing aberrant polarization of immune cells such as T lymphocytes; promoting the recruitment and activation of immunosuppressive cells such as M2 tumor-associated macrophages (TAMs) and Treg cells; and decreasing the cytotoxic function of immune effector cells such as NK cells and cytotoxic T lymphocytes (CTLs) (5).

**Figure 1:**
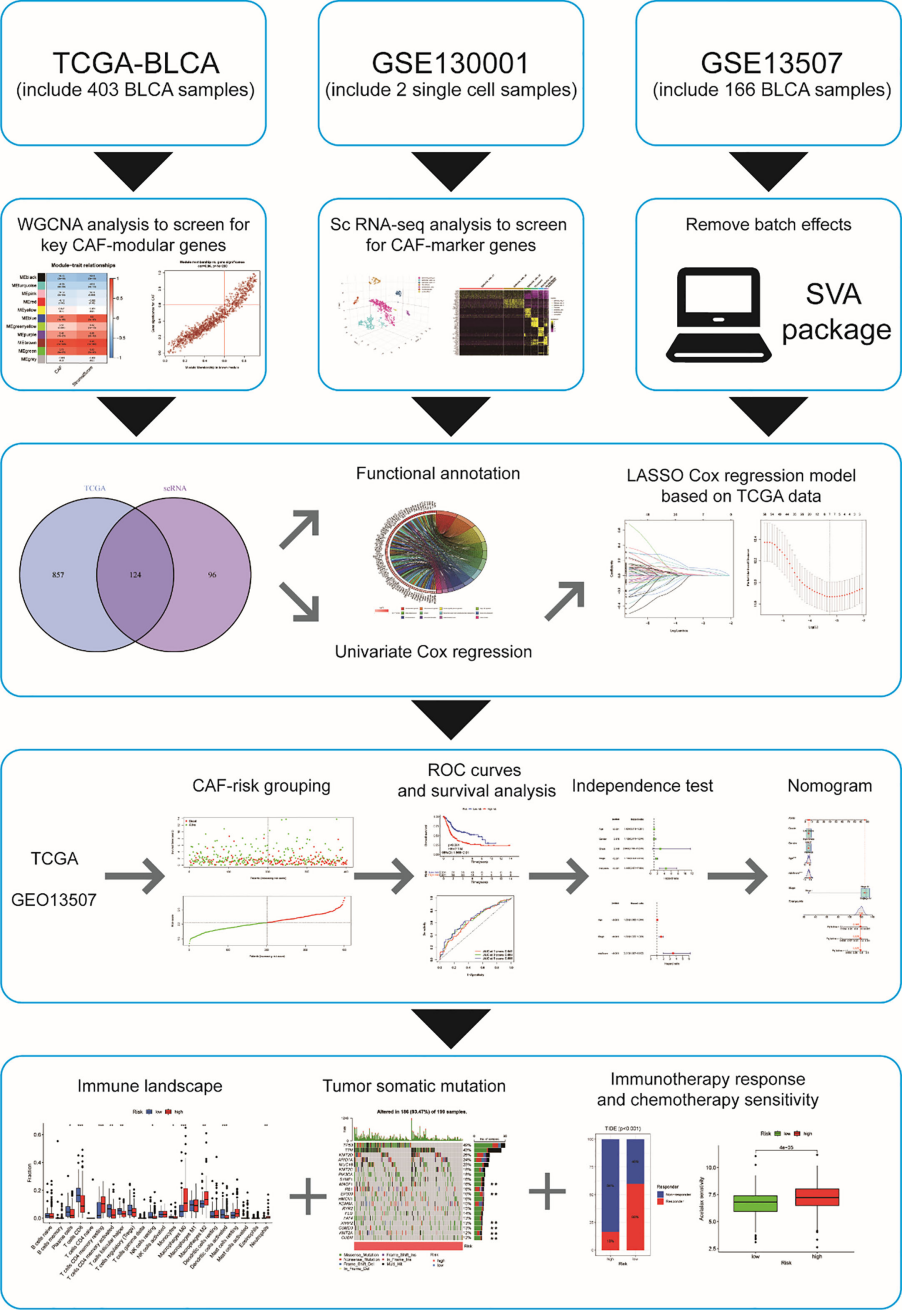
Flowchart of this study p< 0.05, **p< 0.01, and ***p< 0.001.

Despite several investigations on CAF in BCLA, little is known about its systemic properties and their relevance to BCLA prognosis and immunotherapy response. Recently, Bitian et al. (6) identified 15 genes specific to CAF for predicting the proportion of CAF in bladder cancer tissue. Single-cell RNA sequencing (scRNA-seq) allows researchers to examine the heterogeneity of tumors and the cells that surround them at the cellular level. We obtained BLCA scRNA-seq data and transcriptome data (bulk RNA-seq) from publicly available databases based on their findings. Our goal is to find promising CAF-associated polygenic signatures that can predict prognosis, immunotherapy response, and drug sensitivity in BLCA patients. The flowchart of this study is shown in **Figure 1**.

## 2 Materials and methods

### 2.1 Data source

In this study, scRNA-seq files from two muscle-invasive bladder cancer specimens were obtained from the GEO database (http://www.ncbi.nlm.nih.gov/geo) from accession number GSE130001 (7). Clinical information (**Table S1**), transcriptomic data, and somatic mutation data related to BLCA were obtained from The Cancer Genome Atlas (TCGA) database (www.Cancer.gov/), microarray expression data and corresponding clinical data were obtained from the GPL6102 platform for GSE13507 and GSE32894 from the GPL6102 platform GPL6947 in the GEO database (8). TCGA, GSE13507 and GSE32894 were used as the training set and external validation set respectively, after removing batch effects *via* the “SVA” package.

### 2.2 The fraction of CAF in TME

The infiltration of CAF in the TCGA and GSE13507 groups was calculated using bulk RNA-seq-based EPIC (9). Similarly, xCell (10), MCP-counter (11), and ESTIMATE (12) were also used for both groups to predict the infiltration of CAF, as well as the StromalScore. “Surv_categorize” function, was used to calculate the optimal cutpoint value to distinguish between the high and low CAF and StromalScore groups in the TCGA and GSE13507 samples. The “survival” package served to analyze and compare the survival rates of low and high CAF (or StromalScore) by the Kaplan-Meier method to determine whether CAF and/or StromalScore levels correlate with survival in bladder cancer.

### 2.3 Construction of weighted gene coexpression networks

The “WGCNA” package was utilized to construct a weighted gene co-expression network analysis (WGCNA) in the TCGA cohort (13). After filtering the bulk RNA-seq data from TCGA and removing outliers, we constructed Pearson correlation matrices and generated weighted neighbor-joining matrices to emphasize strong correlations and penalize weak correlations. A scale-free network was constructed using the function powerEstimate soft threshold to select the best soft threshold power β = 4. A topological overlap matrix (TOM) was generated. The TOM-based correlation dissimilarity measure was set at a minimum number of genes/modules of 30, resulting in the generation of 11 modules. Next, we performed correlation analysis between modules and EPIC-based CAF infiltration with StromalScore, and modules with the highest correlation coefficients were regarded as candidates for correlation with differentially infiltrated immune cells. After selecting candidate modules, we defined |MM| (|module membership|) > 0.6 and |GS| ( |gene significance|) > 0.6 as the screening criteria for screening key genes in candidate modules.

### 2.4 Single-cell analysis

The scRNA-seq data was then processed with the “Seurat” package. After combining two muscle-invasive bladder cancer specimens using the “merge” function, cells with excluded genes discovered in less than three cells and detected genes detected in fewer than 200 genes were eliminated, with the fraction of mitochondria confined to less than 5%. The scRNA-seq data from high-quality cells were normalized and the “FindVariableFeatures” function looked for highly variable genes for downstream analysis. Principal component analysis (PCA) was then performed on the highly variable genes to identify significant principal components (PCs). After the initial 12 PCs were selected. Cell clustering was then visualized using the t-SNE algorithm using the t-distribution random neighborhood embedding, and the “plotly” package was plotted in 3D. The “FindAllMarkers” function was used to detect marker genes for each cell cluster with a log2-fold change (FC) filter value > 1 and p_val_adj< 0.05. We made statements for each cell cluster based on the following marker genes: Epithelial cells (KRT19, CDH1, EPCAM); Fibroblasts (MMP2, EMILIN1, SFRP2); Myofibroblasts (ACTA2, PDGRB); Endothelial cells (KDR, VCAM1, AQP1, SEMA3G, CLDN5, PLVAP). To improve accuracy, we compared key genes of the WGCNA module, which is highly associated with CAF, with key genes with CAF characteristics.

### 2.5 Functional annotation

To determine the function of key genes and reveal their underlying biological functions and potential mechanisms, we performed Gene Ontology (GO) and Kyoto Gene Encyclopedia and Genomic Pathway Enrichment Analysis (KEGG) using the clusterProfiler R package, p< 0.05 and FDR< 0.05.

### 2.6 Construction and validation of a CAF-related prognostic signature

To obtain CAF-related genes for which prognostic markers could be constructed, univariate Cox regression was performed to examine the correlation between CAF genes and overall survival (OS) in the TCGA dataset. CAF genes with a p-value< 0.05 were considered significant prognostic genes. To minimize the risk of overfitting, we then applied a least absolute shrinkage and selection operator (LASSO) Cox regression model *via* the “glmnet” R package. Based on the LASSO regression coefficients and gene expression, a risk score was calculated for each bladder cancer in the training set under the following equation:


$$riskScore=\sum_{i=1}^{n}\left[Exp\left(genes\right)*\;coefficient\left(genes\right)\right]$$

This was subsequently categorized into high and low CAF risk groups based on the median TCGA CAF-riskScore and generalized to the validation group. Heat maps were generated to visualize the association between CAF-riskScore and candidate genes. The prognostic performance of CAF-riskScore was assessed by time-dependent subject operating characteristic (ROC) curves and survival analysis. Univariate and multivariate Cox regression analyses were performed to determine whether the CAF-riskScore independently served as a significant prognostic indicator. Nomograms were established for the GSE13507 and TCGA cohorts. Calibration curves were used to evaluate the predictive performance of these nomograms.

### 2.7 TME infiltration estimation

The proportion of immune cell infiltration in TCGA samples was calculated using CIBERSORT (14). We examined the differences in the infiltration of immune cells under risk grouping and the correlation of CAF-riskScore with certain cells in TME.

### 2.8 Correlation analysis between CAF riskScore and CAF infiltration

Spearman correlation estimated the relationship between the CAF riskScore and the CAF infiltration predicted by the above algorithm. Heatmaps were plotted by the “GGally” package. Spearman correlations similarly measured the CAF-riskScore and the relationship between candidate genes and known CAF-associated genes, and the “ggplot2” package did the plotting.

### 2.9 GSEA analysis

GSEA analysis was performed on the high CAF group from the TCGA cohort based on the Molecular Signature Database (MSigDB) (c2.cp.KEGG gene sets, hallmark gene sets), using default settings, and the top 5 and P< 0.05 pathways were plotted for each human collection gene sets.

### 2.10 Acquisition of gene mutation information

Mutated genes were calculated using the Tumor Mutation Burden (TMB). The “maftools” R package was used to visualize the top 20 genes with the highest mutation frequency in both groups.

### 2.11 Analyses of immunotherapy and drug screening

TIDE is a technique for assessing the possibility for immune evasion based on transcriptional profiling (15). TIDE allows the assessment of the effectiveness of immune checkpoint blockade for ICI therapy including anti-PD1 and anti-CTLA4 therapies. After using all tumor sample means as normalized controls, we downloaded TIDE estimates for each sample high and low CAF-risk groups of TCGA and GSE13507 cohorts and from the TIDE website (http://tide.dfci.harvard.edu/).“Ggpubr” and “reshape2” package were used for visualization.

Based on expression and sensitivity data from the GDSC2 database, the “oncoPredict” package was used to predict the drug sensitivity of different drugs in the high and low CAF-risk groups (16).

### 2.12 Cell lines and cell culture

All cell lines were purchased from American-Type Culture Collection (ATCC). Human immortalized uroepithelial (SV-HUC-1) SV-HUC-1 cell line was cultured with Ham’s F-12K (HyClone, China)/10% fetal bovine serum (Gibco, Australia) media while BLCA cell lines (5637, T24) were cultured with RPMI 1640 (HyClone, China)/10% fetal bovine serum media. All cells were cultured in an incubator with 5% CO2 at 37°C.

### 2.13 Quantitative real-time polymerase chain reaction (qRT-PCR)

TRIzol reagent (Thermo Fisher Scientific, USA) was used to extract RNA from the cells and the PrimeScriptTM RT Reagent Kit (TaKaRa, Japan) was used for reverse transcription into cDNA, respectively. After all target RNAs have been reverse transcribed to cDNA, relative quantitation by real‐time PCR was performed using TB Green PCT Master Mix (akara, Japan). And then qRT-PCR analysis was performed using a CFX96 real-time PCR system. GAPDH was used for experimental reference. All primers, the sequences of which are listed in **Table S2**, were synthesized by Sangon Biotech (Shanghai).

### 2.14 Statistical analyses

Statistical analysis was performed using R 4.2.0 and GraphPad Prism 8. The data package used for statistical analysis within R was as described above. Correlation matrices were analyzed using Pearson or Spearman for correlation. Survival analysis was performed using the Kaplan-Meier method and Log-rank tests. Comparisons between the two groups were made using the Wilcoxon test. A chi-square test was used for categorical variables. The differences in p-values< 0.05 were considered to be statistically significant.

## 3 Results

### 3.1 Screening for CAF-related genes by WGCNA in BLCA

**Figure 2:**
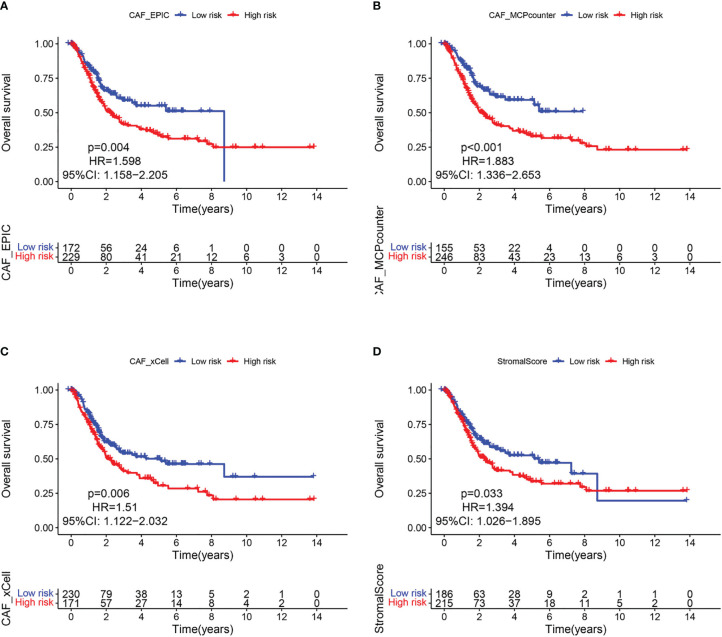
CAF-related survival analysis in the TCGA cohort. Kaplan–Meier survival curves of CAF infiltration evaluated by **(A)** EPIC, **(B)** MCP-counter, **(C)** xCell, and **(D)** StromalScore.

Since CAF is of import in tumorigenesis, it is urgent to further elucidate the relationship between CAF and BLCA prognosis. The EPIC, xCell, and MCPcounter algorithms were used to calculate the amount of CAF cells in TCGA samples. BLCA patients were then divided into high and low fibroblast content groups. Kaplan-Meier analysis showed that the 3 algorithms had a longer survival rate in the low CAF content group than in the high CAF infiltration group (**Figures 2A–C**), thus suggesting that CAF play a major role in BLCA. Similar results were found for the ESTIMATE-based StromalScore (**Figure 2D**), with fibroblasts being an important component of the stromal cells.

**Figure 3:**
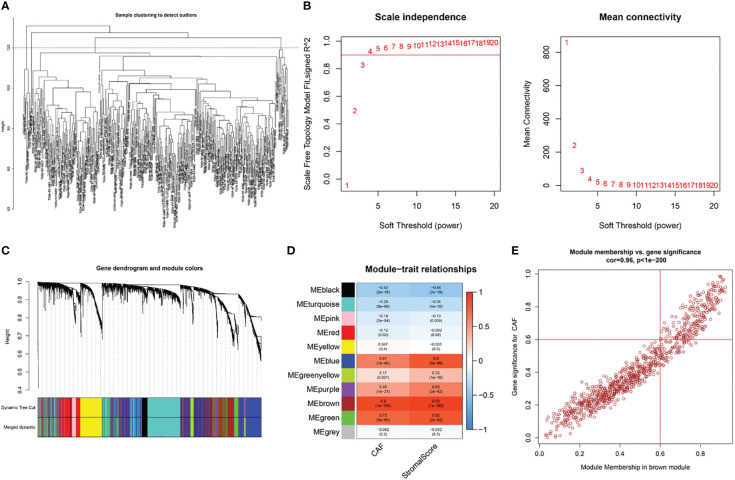
WGCNA of CAF-related genes in TCGA cohort. **(A)** Samples were clustered to detect outliers and sample Samples above the red line were reserved. **(B)** Scale-free topology model fit (left) and mean connectivity (right) for the appropriate soft threshold power. The power selected was 4. **(C)** The cluster dendrogram constructing the gene modules and module merging. **(D)** Correlation analysis between the module and CAF fraction and StromalScore. **(E)** Correlation between GS and MM in the brown module.

Based on this observation, WGCNA was used to identify CAF-associated genes in bladder cancer. Supported by the depth of CAF infiltration in the EPIC package with StromalScore, WGCNA was utilized to identify CAF-associated genes in bladder cancer. First, samples above 120 were removed from the TCGA cohort (**Figure 3A**), selection 4 was chosen as the optimal soft threshold power (no scale R2 = 0.9254) (**Figure 3B**), and WGCNA identified 11 modules as shown in **Figures 3C, D**: of these, the brown module was significantly associated with high levels of CAF cells, (correlation = 0.9, p<0.0001), and the brown module The correlation between GS and MM is a key measure of the quality of gene module construction. After correlating the proportion of brown modules with CAF, the correlation between GS and MM reached 0.96 (**Figure 3E**), suggesting that all genes in the pink modules are specifically expressed by CAF in BLCA and that the expression levels of these genes are not easily influenced by other cells. Therefore, we set more stringent screening conditions of GS correlation > 0.6 and MM correlation > 0.6 and selected 981 key genes for downstream analysis (**Table S3**).

### 3.2 Single cell RNA-seq profiling, clustering, and markers identifications

**Figure 4:**
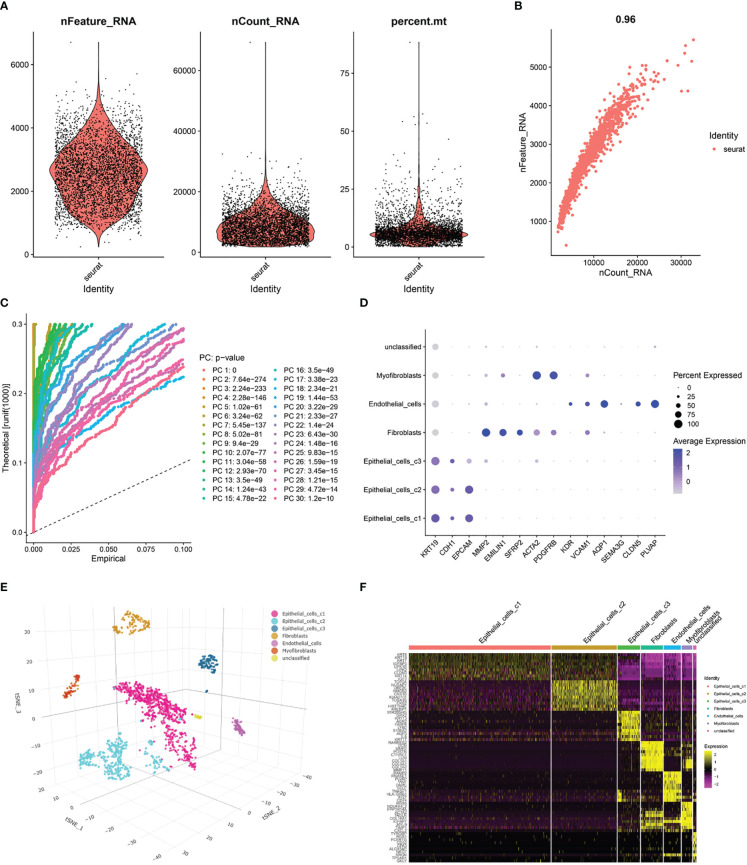
Processing of scRNA-seq data and acquisition of CAF marker genes. **(A)** Quality control of scRNA-seq data of BLCA cells. **(B)** Correlation between the number of genes and the depth of sequencing. **(C)** Principal component analysis (PCA) of the high-quality cells, and the top-30 PCs were displayed. **(D)** Dotplot of marker gene expression of each cell cluster. **(E)** T-SNE plot showing 7 clusters identified by integrated analysis, colored by cell cluster. **(F)** Heatmap of the marker genes with differential expression in each cell cluster.

After pre-processing scRNA-seq data from GSE130001 based on the stringent quality control metrics described, 1623 high-quality cell samples were separated from the 2 found bladder cancer tissues. The number of genes detected (nFeature) and the depth of sequencing (total UMI, nCount), and the percentage of mitochondrial genes (percent.mt) were plotted (**Figure 4A**), with a strong positive correlation between nFeature and nCount and a Pearson correlation coefficient of 0.96 (**Figure 4B**). Subsequently, we used the t-SNE technique on the first 12 major components (**Figures 4C**, **S1**) to visualize the high-dimensional scRNA-seq data and successfully classified the cells into 12 clusters, followed by the following marker genes declared for each cell cluster (**Figures 4D, E**). In addition, the threshold for significantly expressed marker genes in each cluster was logFC > 1, adjPval< 0.05, and the top 10 markedly different genes for each cluster were shown by heatmap (**Figure 4F**). Of these, 220 genes were selected as CAF-related genes (**Table S3**).

### 3.3 GO and KEGG functional annotation analyses of CAF marker genes

**Figure 5:**
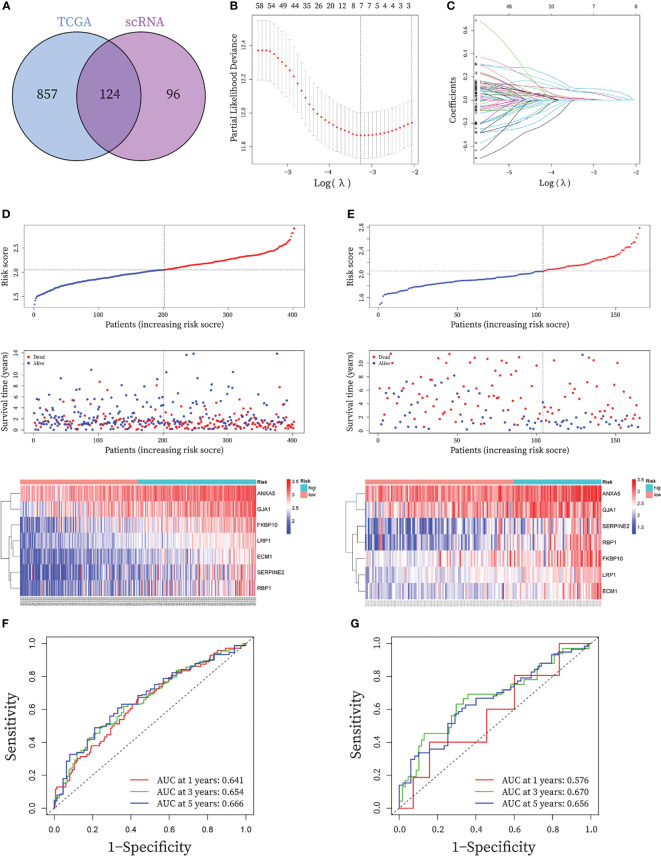
Construction and validation of seven-gene prognostic CAF signature for BLCA patients. **(A)** The Venn graph of the CAF-genes obtained from WGCNA key genes in TCGA cohort and scRNA-seq marker genes. **(B)** To determine the penalty term parameter (λ), partial likelihood deviations are displayed. **(C)** The Lasso regression coefficient profiles showing the change in coefficients for different variables with the λ penalty **(D-E)** Risk plot distribution, survival status of patients, and heatmap of expression of seven CAF-genes in the **(C)** TCGA cohort and the **(D)** GEO cohort. **(F-G)** Receiver operating characteristic (ROC) curves for the CAF signature in the **(E)** TCGA cohort and the **(F)** GEO cohort.

**Figure 6:**
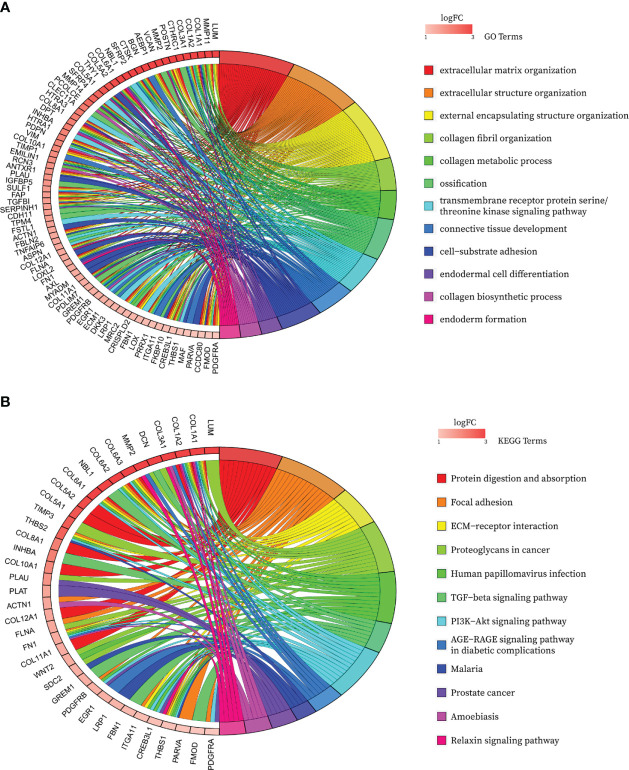
Bubble map of functional annotation analyses of 124 CAF marker genes with the **(A)** GO database and the **(B)** KEGG database.

After de-intersecting the key genes obtained from WGCNA and the CAF marker genes obtained from the single-cell analysis, 124 candidate CAF-related genes were obtained for subsequent analysis (**Figure 5A**; **Table S3**). As shown in **Figure 6A**, enriched GO terms were significantly enriched mainly in extracellular matrix organization, extracellular structure organization, external encapsulating structure organization, transmembrane receptor protein serine/threonine kinase signaling pathway and connective tissue development. **Figure 6B** shows the top 12 enriched KEGG pathways, which mainly include Focal adhesion, ECM-receptor interaction, Proteoglycans in cancer, TGF−beta signaling pathway and PI3K Akt signaling pathway. These enrichment terms enhance the reliability of the marker gene screen.

### 3.4 Construction and validation of seven-gene prognostic CAF signature

In the TCGA cohort, a total of 80 genes exhibited P< 0.05 by entering the 124 CAF marker genes mentioned above into univariate Cox regression analysis (**Table S4**). The final LASSO regression analysis identified seven prognostic marker genes: LDL Receptor Related Protein 1 (LRP1), Annexin A5 (ANXA5), Serpin Family E Member 2 (SERPINE2), Extracellular Matrix Protein 1 (ECM1), Retinol Binding Protein 1 (RBP1), Gap Junction Protein Alpha 1 (GJA1), FKBP Prolyl Isomerase 10 (FKBP10) (**Figures 5B, C**). CAF-riskScores were computed for each sample in TCGA based on the coefficients (**Table S3**) and the expression of prognostic genes. BCLA patients in the training set were then divided into low and high-risk groups based on the median riskScore, (**Figure 5D**), and extended to the validation group (**Figure 5E**). To assess the performance of the risk model, ROC curves were plotted and the area under the ROC curve (AUC) was 0.641, 0.654, and 0.666 for 1, 3, and 5 years in the training group, respectively (**Figure 5F**), and 0.576, 0.670 and 0.656 for 1, 3 and 5 years in the validation group (**Figure 5G**), respectively. In the GSE32894 database, CAF-riskScores also has good predictive performance. AUC for 1, 3 and 5 years was 0.687, 0.669 and 0.697 (**Figure S2**).

### 3.5 Evaluation of the predictive capability of CAF signature

**Figure 7:**
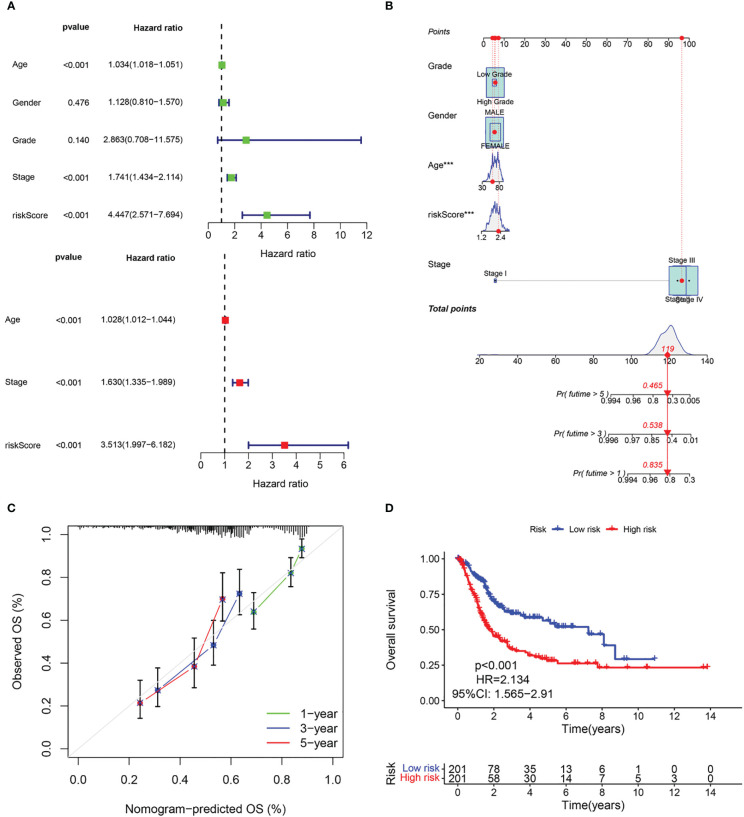
Evaluation of the predictive capability of CAF signature based the TCGA cohort. **(A)** Forest maps of the univariate and multivariate Cox regression analysis between the CAF-riskScore and clinical characteristics. **(B)** Nomogram predicting the survival rate at 1, 3, 5 years for BLCA patients, and ***p< 0.001. **(C)** Calibration plots for the nomogram. **(D)** Kaplan–Meier survival curve for the CAF-risk subtypes.

**Figure 8:**
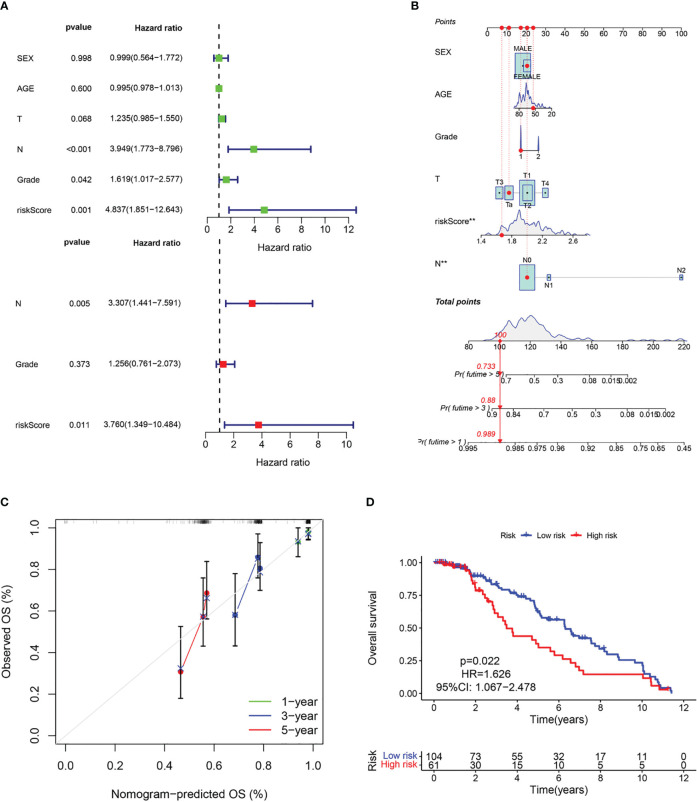
Evaluation of the predictive capability of CAF signature based the GEO cohort. **(A)** Forest maps of the univariate and multivariate Cox regression analysis between the CAF-riskScore and clinical characteristics. **(B)** Nomogram predicting the survival rate at 1, 3, 5 years for BLCA patients, and **p< 0.001. **(C)** Calibration plots for the nomogram. **(D)** Kaplan–Meier survival curve for the CAF-risk subtypes.

The above results suggest that the CAF-riskScore could be a promising prognostic biomarker for patients with bladder cancer. Therefore, to determine whether CAF-riskScore could be used independently as a prognostic indicator, we performed univariate and multivariate Cox regression analyses in the TCGA and GSE13507 cohorts. The results showed that the riskScore (HR=3.513, 95% CI:1.997-6.182, P<0.001), TNM Stage (HR=1.630, 95% CI:1.335-1.989, P<0.001), and age (HR=1.028, 95% CI:1.012-1.044, P<0.001) were independently associated with OS (**Figure 7A**), and in the GSE13507 cohort (**Figure 8A**), riskScore (HR=3.760, 95% CI:1.349-10.484, P=0.011) was consistently validated as an independent prognostic indicator. To predict OS rates for patients with bladder cancer in the TCGA (**Figure 7B**) and GSE62254 (**Figure 8B**) cohorts, we created a predictive nomogram for clinicians including the CAF-riskScore. Calibration curves showed that the model predictions for 1-, 3- and 5-year OS probabilities were consistent with the ideal predictions (grey line) in all datasets (**Figures 7C**, **8C**). These results suggest that the nomogram model can be invoked as a reliable tool for predicting OS in bladder cancer patients. Two-by-two comparisons of OS in different risk groups were investigated by log-rank tests. Kaplan-Meier curves showed that the group with high CAF-risk tended to have significantly detrimental survival outcomes compared to the low CAF-risk group (TCGA cohort, hazard ratio (HR) = 2.134, 95% CI:1.565 -2.91, log-rank P<0.001, **Figure 7D**; GSE13507 cohort, HR=1.626, 95% CI:1.607-2.470, log-rank P=0.022, **Figure 8D**; GSE32894 cohort, HR=2.927, 95% CI:1.26-6.801, log-rank P=0.009, **Figure S2D**). Notably, the CAF-risk grouping for GSE13507 was provided by the median CAF-riskScore of the TCGA cohort.

### 3.6 CAF signature-related TME infiltration landscape

**Figure 9:**
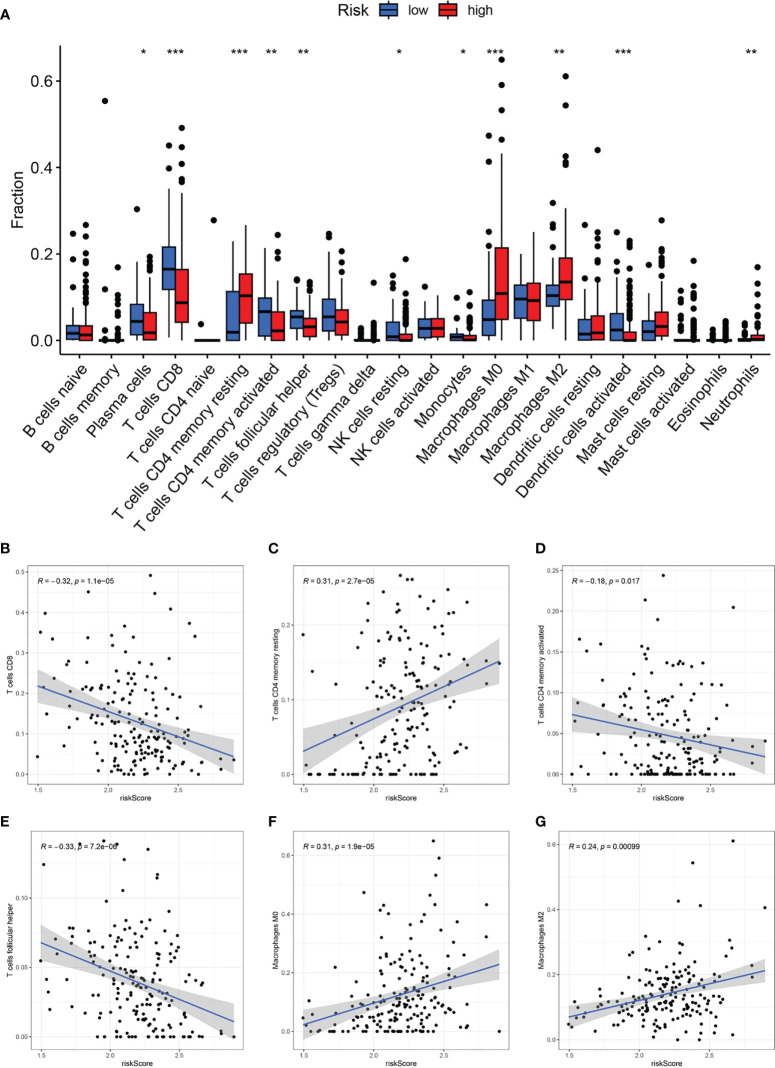
CAF-signature related immune landscapes. **(A)** The bar graph of difference in composition of the 22 types of immune cells between two CAF-risk subtypes, *p< 0.05, **p< 0.01, and ***p< 0.001. **(B-H)** The correlation between the CAF-riskScore and **(B)** CD8 T cells, **(C)** resting CD4 memory T cells, **(D)** activated CD4 memory T cells, **(E)** follicular helper T cells, **(F)** M0 Macrophages and **(G)** M2 Macrophages.

Based on the Cibersort algorithm, we investigated the correlation between CAF-riskScore and TME components at the bulk RNA-seq level. As shown in **Figure 9A**, according to risk grouping, in terms of immune cells, we found that the high CAF group had significantly higher resting CD4 memory T cells, M0 Macrophages, M2 Macrophages, and Neutrophils, than the low CAF group. In contrast, Plasma cells, CD8 T cells, activated CD4 memory T cells, follicular helper T cells, resting NK cells, Monocytes, and activated Dendritic cells, were significantly higher in the high CAF group. Using correlation graphs to identify the correlation between CAF-riskScore and TME components. Notably, as the riskScore increased, the infiltration of CD8 T cells, CD4 memory T cells, and follicular helper T cells gradually decreased, while M0 Macrophages and M2 Macrophages gradually replaced some of the above cells (**Figures 9B-G**).

### 3.7 Relationship of the fraction of CAF with CAF signature

**Figure 10:**
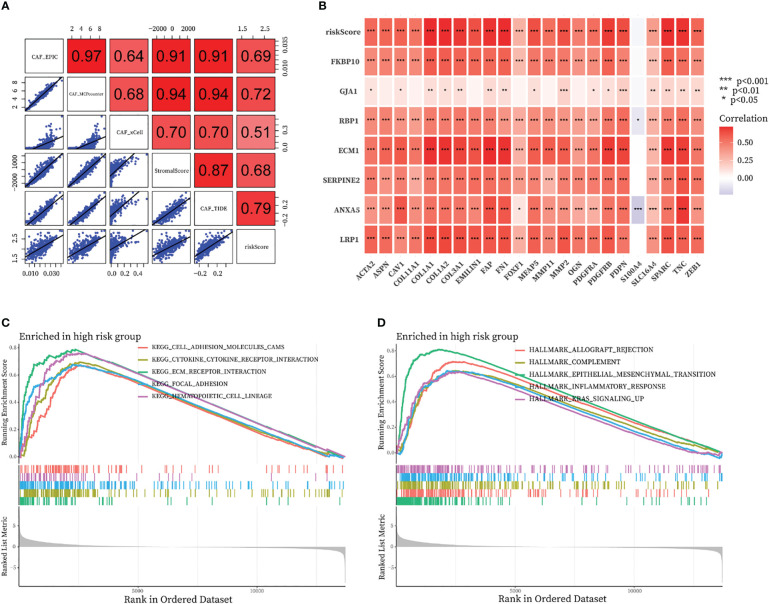
Relationship of the fraction of CAF with CAF-signature and results of GSEA analysis in TCGA cohort. **(A)** Correlation analysis between CAF-riskScore and CAF infiltration based on EPIC, xCell, MCPcounter, ESTIMATE, and TIDE algorithms. **(B)** Relationship between know CAF related gene expression and the expression of the CAF-riskScore and seven CAF-signature component gene. **(C, D)** GSEA analysis of high CAF-risk subtype based on **(C)** KEGG gene sets and **(D)** hallmark gene sets.

By running the EPIC, xCell, MCPcounter, ESTIMATE, and TIDE algorithms, we investigated the correlation between CAF-riskScore and the fraction of CAF in the bulk RNA-seq level. As shown in **Figure 10A**, the riskScore obtained from the CAF model was highly positively correlated with the degree of CAF infiltration obtained from EPIC, MCPcounter, ESTIMATE, and TIDE. We also discussed the relationship between CAF-related gene expression and the expression of the CAF-riskScore and its component genes. In the TCGA cohort, CAF-riskScore was positively correlated with all CAF-related genes except S1000A4, and p<0.05 (**Figure 10B**).

### 3.8 GSEA functional annotation analyses of CAF signature

We then investigated the functional pathways in this model by GSEA analysis. Patients in the TCGA cohort were divided into high-CAF and low-CAF groups according to the above approach. We found immune and extracellular communication features in the engaged CAF group, including cell adhesion molecules, cytokine-cytokine receptor interaction, ECM-receptor interaction, and focal adhesion (**Figure 10C**). In addition, several hallmark gene sets were also significantly enriched in the high CAF-risk group, including allograft rejection, complement, epithelial mesenchymal transition, inflammatory response, and kras signaling up (**Figure 10D**).

### 3.9 Relationship between CAF signature and somatic variation

**Figure 11:**
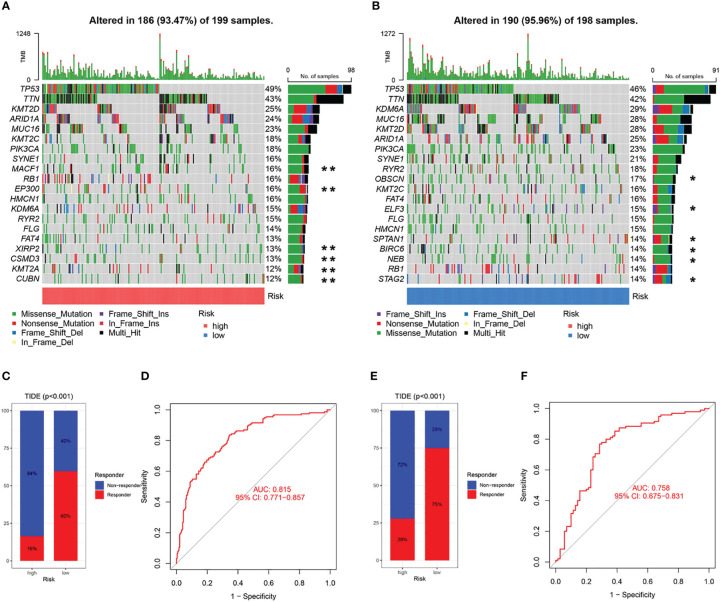
Characteristics of CAF-signature with tumor somatic mutation and immunotherapy response. **(A, B)** Waterfall plots of top 20 mutated genes in the TCGA cohort in the high CAF-risk subtype **(A)** and low CAF-risk subtype **(B)**. **unique mutation in the high CAF-risk subtype; *unique mutation in the high CAF-risk subtype. **(C-F)** TIDE analysis for predicting the possibility of clinical response to immunotherapy in the **(C, D)** TCGA cohort and the **(E, F)** GEO cohort.

The 20 most commonly mutated genes in the high (**Figure 11A**) and low (**Figure 11B**) CAF-risk groups are shown as waterfall plots, with some genes present in both groups, including TP53, TTN, KMT2D, ARID1A, MUC16, KMT2C, PIK3CA, SYNE1, RB1, HMCN1, KDM6A, RYR2, and FLG. MACF1, EP300, FAT4, XIRP2, CSMD3, KMT2A, and CUBN mutations were uniquely present in the high CAF-risk group, while OBSCN, ELF3, SPTAN1, BIRC6, NEB, and STAG2 mutations were uniquely seen in the low CAF-risk group.

### 3.10 Analyses of immunotherapy response and drug screening of CAF signature

**Figure 12:**
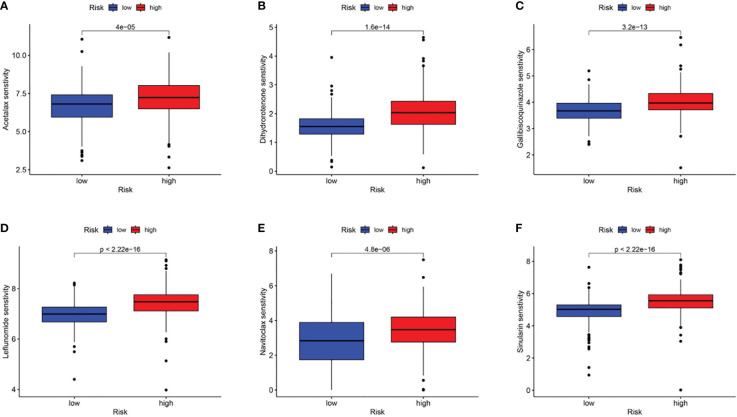
Association of CAF-riskScore with chemotherapy sensitivity. Estimated sensitivity for **(A)** acetalax, **(B)** dihydrorotenone, **(C)** gallibiscoquinazole, **(D)** leflunomide, **(E)** navitoclax and **(F)** sinularin in high and low CAF-risk subtypes.

Subsequently, using the TIDE online algorithm, we predicted the probability of response to immune checkpoint inhibitors in both datasets. In the TCGA cohort, as shown in **Figure 10C**, only 16% of patients in the high CAF-risk group had a response to immunotherapy, in the low CAF-risk group this was 60%, (**Figure 11C**, chi-square test, p<0.001). We then used the ROC curve to assess the relationship between the CAF-riskScore and the two groups of responders versus non-responders, with an AUC of 0.815, 95% CI:0.771-0.857. In the GSE13507 cohort (**Figure 11D**), again, also in the low-CAF risk group, more patients responded to immunotherapy (**Figure 11E**, 75% vs 28%), and AUC was 0.758 (**Figure 11F**). The drug sensitivity of each patient was then calculated by the oncoPredict algorithm to seek different chemotherapeutic agents for the CAF-related high and low CAF-risk groups. Based on the GDSC2 cancer cell line database, we found that higher CAF-riskScore increased TCGA in 6 anticancer drugs (acetalax, dihydrorotenone, gallibiscoquinazole, leflunomide, navitoclax, sinularin) sensitivity (**Figures 12A-F**).

### 3.11 Expression of seven prognostic CAF genes in BLCA

**Figure 13:**
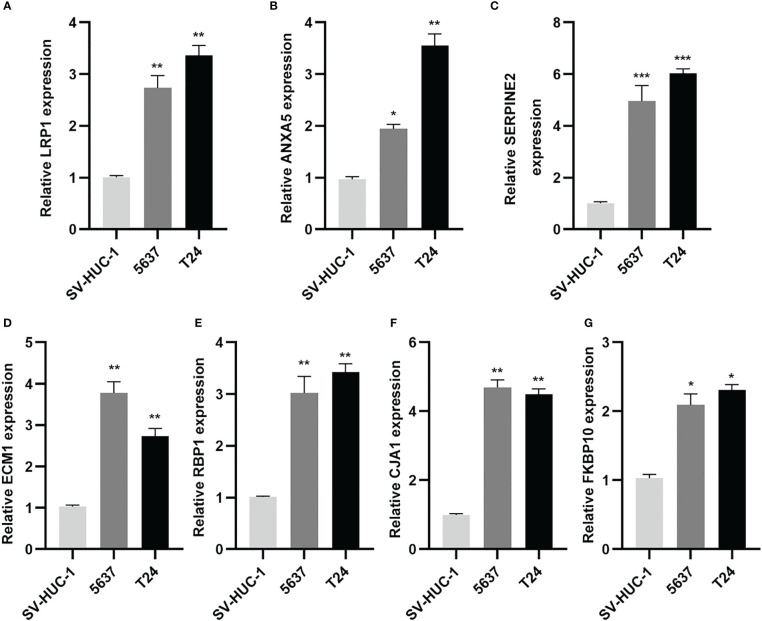
CAF-related genes were highly expressed in BLCA lines. **(A-G)** Relative expression of mRNAs (LRP1, ANXA5, SERPINE2, ECM1, RBP1, GJA1, FKBP10) in 2 BLCA cell lines (5637, T24) and SV-HUC-1 cell line was measured using qRT-PCR. *p< 0.05, **p< 0.01, and ***p< 0.001.

Subsequently, the qRT-PCR measured the seven genes level (**Figure 13**). Thus, all the expression of CAF genes was higher based on the normal cell line in BLCA cell line.

These results suggest that while a high CAF-riskScore is associated with increased sensitivity to some chemotherapies, immunotherapy may be more effective in bladder cancer patients with low CAF-riskScore.

## 4 Discussion

CAF has received increasing attention in the last decade due to its key role in tumorigenesis and progression (17). Through multiple pathways, activated CAF can promote tumor growth, invasion, and metastasis, as well as ECM remodeling (18), and even interact with other cells in the TME to form an immunosuppressive loop, further enhancing immunosuppression in the TME (5). Specifically in BLCA, CAF promotes the Wnt signaling pathway in bladder cancer cells through paracrine IL1β, thereby enhancing their proliferation and invasion (19). Also, previous studies have demonstrated that by modifying CAF-derived exosomes, we enhance the chemosensitivity of bladder cancer cells (20). However, the clinical application remains challenging due to the lack of effective targeting biomarkers, which prompted us to investigate novel CAF markers for bladder cancer.

We found that TCGA samples with high CAF content had a significantly lower prognosis than those with low CAF content, suggesting an association between CAF and bladder cancer prognosis. Then, 124 CAF-associated genes were obtained by crossing CAF modular genes screened from TCGA with fibroblast marker genes screened from the GEO database. Following univariate regression and LASSO regression analysis, seven prognosis-related genes (LRP1, ANXA5, SERPINE2, ECM1, RBP1, GJA1, FKBP10) were screened to be able to predict the construct prognostic characteristics of bladder cancer patients and to evaluate TME mesenchymal and fibroblast components and treatment response. In this study, we have demonstrated that CAF-related features are independent risk factors associated with OS. To improve the predictive effectiveness of this feature for clinical application, we subsequently constructed and validated the clinical utility of a nomogram for predicting OS based on clinical features and CAF-riskScore.

Of these biomarkers in this well-established CAF signature, LRP1, GJA1 is most closely connected with CAF. LRP1, for example, regulates fibroblast migration and is critical in skin wound healing (21). The capacity of LRP1 to induce endocytosis and transmit cell signals plays many functions in carcinogenesis and tumor growth (22). LRP1 enhances ESCC cell migration and invasion in esophageal squamous cell carcinoma (ESCC) through interaction with CAF-derived PAI-1 and is eventually linked with a poor prognosis in ESCC patients (23). Pro-cath-D interacts with LRP1 on the fibroblast surface, altering TME in breast cancers (24). LRP1 has also been shown to have predictive significance in metabolism-related genes in bladder cancer (25). GJA1, also known as Cx43, is highly expressed in bladder smooth muscle cells and is implicated in subbasal membrane gap junctions, where mesenchymal afferent and motor impulses are integrated (26, 27). GJA1 deletion fibroblasts had a considerably stronger inflammatory response to cytokine induction than GJA1 wild-type fibroblasts (28). GJA1 plays a complicated, or even conflicting, function in malignancies. We also discovered that Cx43 protein inactivation contributed to malignant tumor angiogenesis (29), although in glioblastoma, GJA1 acted as a tumor invasion promoter (30). This shows that further research is required to fully confirm the predictive usefulness of GJA1 in BLCA patients.

Poor prognosis in cancer patients has also been linked to overexpression of ANXA6. In pancreatic ductal adenocarcinoma (PDA) cells *in vitro*, loss of ANXA6 in CAF altered the development of ANXA6, LRP1, and TSP1 complexes, suppressing PDA and metastasis (31). ANXA5 expression was shown to be considerably higher in individuals with gastric cancer (32), and ANXA5 polymorphism pairs were found to alter glioma susceptibility and prognosis (33). Furthermore, SERPINE2 was identified as a CAF-associated gene in our investigation. In BLCA, SERPINE2 is linked with a poor prognosis (34). SERPINE2 boosts the *in vivo* invasiveness of its highly metastatic cancer cells *via* LRP1-mediated induction of MMP9 (35), and SERPINE2 indirectly enhances the invasive potential of cancer cells in PDA by activating surrounding stromal cells (36). Following ATF3 expression, SERPINE2 causes the improvement of cell colony-forming capacity and is thus involved in the control of cancer growth (37). Different cell types’ signaling pathways are impacted by SERPINE2 activities (35). SERPINE2 increased angiogenesis and lymphangiogenesis in oral squamous carcinoma (OSCC) cells, whereas knocking down SERPINE2 decreased cell proliferation and invasion (38). SERPINE2 increased osteosarcoma cell growth and treatment resistance (39).

It is well recognized that ECM1 is closely related to the emergence of BLCA, even as a participant in tumor angiogenesis (40). High ECM1 expression is related with unfavorable clinicopathological characteristics and a bad prognosis, and it may potentially be employed as an urine biomarker in BLCA patients (41). The knockdown of ECM1 has a major impact on BLCA proliferation, migration, and invasion (42). Integrin X2 and the AKT/FAK/Rho/cytoskeleton signaling pathways are implicated in the promotion of ovarian cancer by the bioactive recombinant ECM1 (ECM1a) subtype (43). All of the above research suggests that ECM1 regulates communication between BLCA cells. RBP1, which is involved in vitamin A metabolism, can be used as a marker for synthetic smooth muscle cells (44) and was used to identify various subpopulations of fibroblasts in cirrhotic patients (45). RBP1 influences the autophagy mechanism in OSCC cells, which has a pro-carcinogenic effect in OSCC (46). It has been demonstrated that FKBP10 expression contributes to the development of colorectal (47), lung (48), renal cell (49), and stomach cancers (50). Through interactions with external mesenchymal receptors and cell adhesion molecules, a different recent study hypothesizes that FKBP10 and other proteins may be closely related (51). This is consistent with our GSEA results (**Figure 7C**).

With increasing evidence, CAF is an important element in suppressing the anti-tumor immune response in TME. Our results demonstrated that the CAF-signature may be used to evaluate a patient’s prognosis for bladder cancer and that a high CAF-riskScore is a signal of ECM remodelling activation. CAF-riskScore was also shown to be strongly related with TGF-beta signaling pathway activation and PI3K Akt signaling pathway activation, all of which are characteristic markers of fibrogenesis and immunosuppression (52–54). Therefore, we investigated the association between CAF-riskScore and immune infiltration, as well as the feasibility of employing it as a biomarker of immunotherapy response in this study. Our results showed CD8 T cells were replaced by follicular helper T cells in the high CAF-risk group, while CD4 memory T cells went from being active to resting. These suggest that in BLCA, CAF causes a pro-oncogenic phenotypic shift in T cells and suppresses the activity of effector T lymphocytes. Thus, the TIDE algorithm showed that patients with CAF-risk bladder cancer were more likely to be unresponsive to anti-PD1 and anti-CTLA4 therapies in both the training and test groups. Mouse breast cancer models have shown that CAF abundance is associated with reduced infiltration of CD8+ T cells and ICB insensitivity (55). Furthermore, single-cell sequencing also revealed that CAF has a pro-proliferative role and that, beside bladder cancer tumor cells, inflammation-associated CAF (iCAF) recruits monocytes to undergo M2 polarisation, contributing to the formation of an immunosuppressive microenvironment (56), in line with our findings. At the same time, CAF interact with cancer cells to construct a remodeled ECM for the infiltration of tumor-killing immune cells (57) and express different calmodulin to promote tumor cell migration and invasion (58). Interestingly, the remodeled ECM may act as a physical barrier to the delivery of anticancer drugs to solid tumors by forming a dense barrier, with CAF promoting chemoresistance in part through the secretion of ECM proteins (59, 60). However, the oncoPredict algorithm shows that patients with CAF high-risk bladder cancer are more sensitive to some chemotherapeutic agents than patients with CAF low-risk. We hypothetically combine conventional chemotherapy with CAF-targeted immunotherapy to improve TME suppression and reawaken T-cell responses in high CAF-risk tumors (61). However, further clinical trials are required for the realization of synergistic therapies. In addition, treatment with the angiotensin-II receptor blocker (ARB), colesartan, has been shown to reduce TGF-β pathway activation in CAF, leading to reduced connective tissue formation, increased drug delivery and increased immunotherapeutic efficacy (62). It is also noteworthy that stimulated by chemotherapeutic agents, CAF transmits exogenous signals that promote cancer cell survival during or after chemotherapy and promote tumor re-progression (63).

There are various limitations to our study. First, we used retrospective data from three available datasets to develop CAF clusters and a CAF-based risk signature for our study. Therefore, additional prospective validation of a larger sample size of independent and multicenter cohorts of BLCA patients are required to demonstrate the stability of the CAF-risk signature. Second, for clinical usage, cross-validation at the proteome level is required.

## 5 Conclusions

In this study, a CAF-related prognostic signature was constructed to this predict the prognosis of BLCA patients. This prognostic signature also shedded light on the TME landscape, predicted responsiveness to anti-PD1 immunotherapy and provided potential targets for BLCA treatment.

## Data availability statement

The datasets presented in this study can be found in online repositories. The names of the repository/repositories and accession number(s) can be found below: https://figshare.com/articles/dataset/row-data_zip/22227037.

## Author contributions

Research design: YL, JJ, and YZ. Data collection and analysis: YL and YZ. Manuscript preparation: YL, JJ, YZ, and ZC. Chart preparation: YL, JJ, and XL. Revisions: JJ, LW, XL, and ZC. All authors contributed to the article and approved the submitted version.
