# Supplementary material for: Construction of cancer- associated fibroblasts related risk signature based on single-cell RNA-seq and bulk RNA-seq data in bladder urothelial carcinoma
Source: Front Oncol. 2023 Apr 14;13:1170893. doi: 10.3389/fonc.2023.1170893 (PMC10140328; doi:10.3389/fonc.2023.1170893)
Supplement: Supplementary file 1 [file DataSheet_1.zip › Supplementary_Material.docx]

Supplementary Material

# Supplementary Data

We have uploaded all of our raw data to Figshare with the DOI: 10.6084/m9.figshare.22227037

# Supplementary Figures and Tables

Figure-S1 T-SNE plot in 3D showing 7 clusters identified by integrated analysis, colored by cell cluster.

FIGURE S2 validation of seven-gene prognostic CAF signature for BLCA patients.

(A-B) Risk plot distribution, survival status of patients, and heatmap of expression of seven CAF in the GSE32894 cohort.

(C) Kaplan–Meier survival curve for the CAF-risk subtypes.

(D) Receiver operating characteristic (ROC) curves for the CAF signature in the GSE32894 cohort.

Table-S1 Clinical features of the BLCA patients included in this study.

Table-S2 The sequences of all primers.

Table-S3 CAF-genes obtained from different way and cox coefficients distribution of the seven CAF-genes

Table-S4 Univariate Cox regression analyses for 80 CAF-genes related to overall survival ground on the TCGA dataset.
