# Supplementary figures and images for: Construction of cancer- associated fibroblasts related risk signature based on single-cell RNA-seq and bulk RNA-seq data in bladder urothelial carcinoma
Source: Front Oncol. 2023 Apr 14;13:1170893. doi: 10.3389/fonc.2023.1170893 (PMC10140328; doi:10.3389/fonc.2023.1170893)

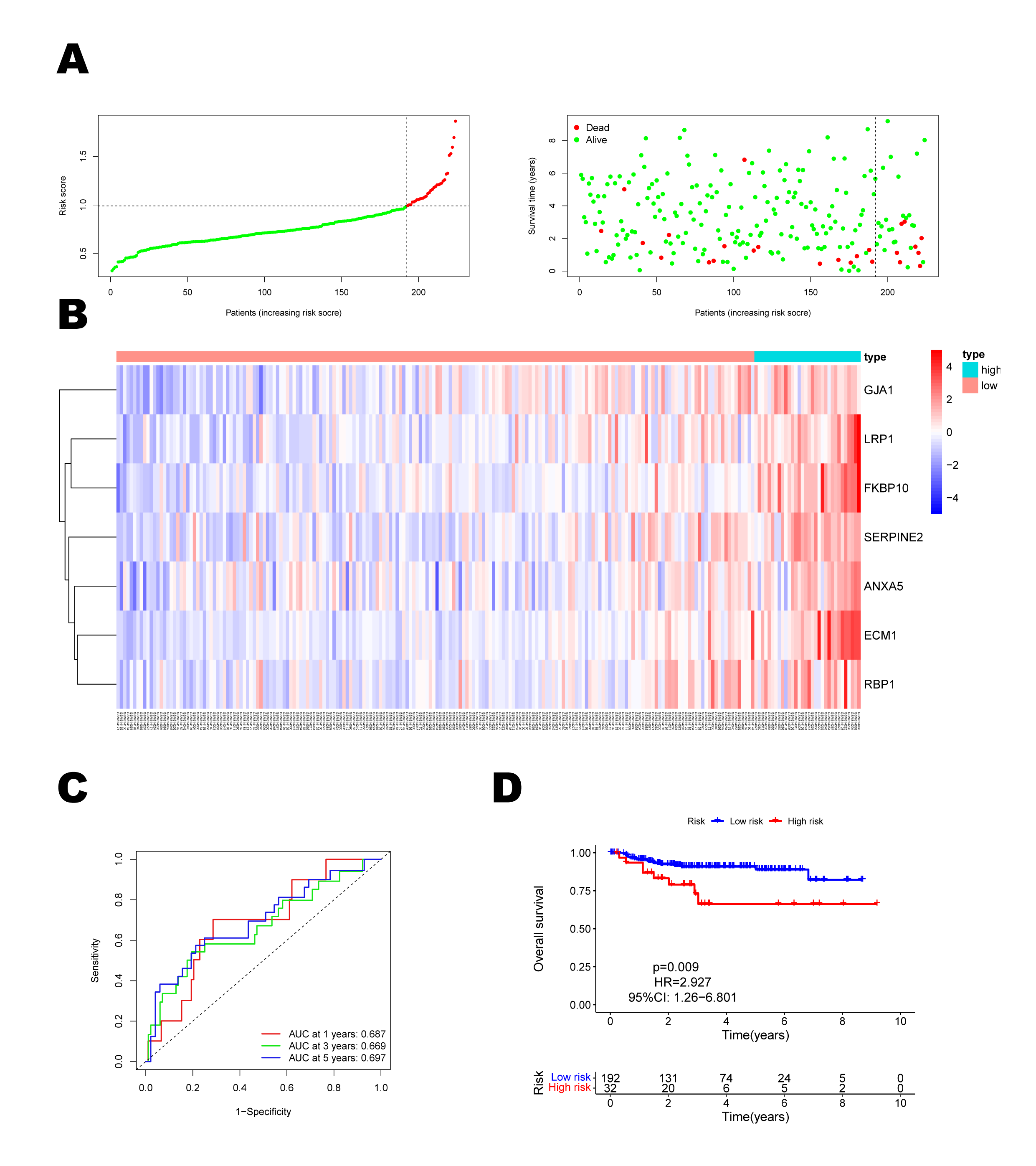

Supplement: Supplementary file 1 [file DataSheet_1.zip › Figure-S2.tif]
